# MXene-Based Ink Design for Printed Applications

**DOI:** 10.3390/nano12234346

**Published:** 2022-12-06

**Authors:** Zahra Aghayar, Massoud Malaki, Yizhou Zhang

**Affiliations:** 1 Metallurgy and Materials Engineering, Iran University of Science and Technology, Narmak, Tehran 16846-11314, Iran; 2 Department of Mechanical Engineering, Isfahan University of Technology, Isfahan 84156-83111, Iran; 3 Institute of Advanced Materials and Flexible Electronics (IAMFE), School of Chemistry and Materials Science, Nanjing University of Information Science and Technology (NUIST), Nanjing 210044, China

**Keywords:** MXene, ink, printing, 2D material

## Abstract

MXenes are a class of two-dimensional nanomaterials with a rich chemistry, hydrophilic surface and mechano-ceramic nature, and have been employed in a wide variety of applications ranging from medical and sensing devises to electronics, supercapacitors, electromagnetic shielding, and environmental applications, to name a few. To date, the main focus has mostly been paid to studying the chemical and physical properties of MXenes and MXene-based hybrids, while relatively less attention has been paid to the optimal application forms of these materials. It has been frequently observed that MXenes show great potential as inks when dispersed in solution. The present paper aims to comprehensively review the recent knowledge about the properties, applications and future horizon of inks based on 2D MXene sheets. In terms of the layout of the current paper, 2D MXenes have briefly been presented and followed by introducing the formulation of MXene inks, the process of turning MAX to MXene, and ink compositions and preparations. The chemical, tribological and rheological properties have been deeply discussed with an eye to the recent developments of the MXene inks in energy, health and sensing applications. The review ends with a summary of research pitfalls, challenges, and future directions in this area.

## 1. Introduction

In the context of printing electronics, printing is commonly employed to precipitate a suspension/solution called ink containing an electrochemical active ingredient onto a substrate of a different kind. Inks usually contain additives such as adhesives, surfactants, and rheology modifiers. The recent boom in the internet of things (IoT) and portable electronics has strongly stimulated the design of advanced and miniatured devices [1,2,3]. Recently, nano-inks, i.e., the inks with nano-scale additives, have been utilized in many printing applications owing to great personalization, reduced wastes, digitalization, improved productivity, scalability, and high performance, among others [1,4,5,6].

To date, many attempts have been performed to produce nano-inks with targeted properties. Graphene [7,8], molybdenum disulfide [9], and black phosphorus [10] inks have been reported as the nano-additives of many inks. The mentioned nano-materials are mostly hydrophobic, and their homogenous dispersions in a given ink may sometimes be problematic; as a result, a third-party agent such as a surfactant or a secondary solvent material is commonly added to most of the printable inks in an attempt to improve their homogeneity and to tune the final rheological property, concentration, conductivity, etc., reaching the desired ink [8,11,12]. Conductive additives like silver nanoparticles [13], graphene sheets [14], gallium [15], etc., have already been used for years to make high-performance conductive inks, and yet efforts in this area are continuing.

A new family of 2D few-atoms-thick layers of transition metal carbides, nitrides, or carbonitrides, called MXenes, has recently drawn heated attention due to special properties like hydrophilicity, mechano-ceramic nature and excellent electrical conductivity, making them an ideal additive for conductive inks, hybrids, or nanocomposites [16,17]. To date, MXenes have successfully been employed in next-generation batteries [18], electromagnetic insulations [19], high-strength composite materials [20], biomaterials [21], and energy and environmental applications [22,23], to name a few. The first member of MXenes, Ti_3_C_2_T_x_, is a carbon-based laminate synthesized through a selective etching process by the Gogotsi group [24] at Drexel University in 2011. Surface terminations such as -O, -OH, and -F commonly appear on the MXene surface [25] and endow a hydrophilicity behavior without sacrificing key properties such as the mobility of charge carriers or conductivity of MXenes [26]. Moreover, the aforesaid functional groups have a significant effect on the physical and chemical properties [27]. 

**Figure 1:**
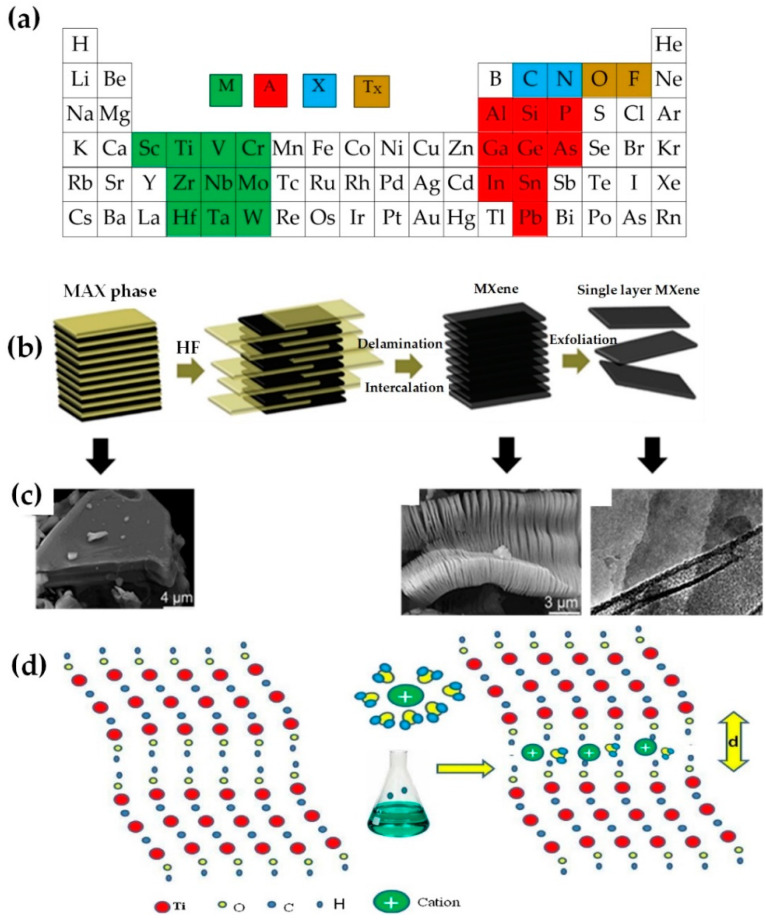
(**a**) Different MAX-phase atoms, (**b**) Top-down synthesis steps of MXene nanosheets from the parent MAX-phase, (**c**) Electron microscope images before and after etching, and exfoliated MXene, reprinted with permission from [28], (**d**) Ions intercalation and delamination in Ti_3_C_2_T_x_ layers, reprinted with permission from [29].

Using a selective etching approach, Figure 1 demonstrates how element A is corroded from a three-dimensional MAX-phase, and a layered accordion structure is then left. The outer surfaces of each layer are covered with functional groups like O, F, or OH. 

After etching, the MAX-phase material (M_n+1_AX_n_T_x_) becomes sheet-structured MXenes (M_n+1_X_n_T_x_) wherein the number n varies from one to four, and T_x_ represents the surface termination groups; M a transition metal, A an element of the group of thirteen or fourteen periodic tables, and X carbon or nitrogen (Figure 1). The type and amount of functional groups depend on the etching path and its conditions; owing to the mentioned functionalities, MXene usually has excellent hydrophilic properties. After the completion of etching and weakening the forces between the MXene layers, the layered particles are delaminated and dispersed in their solvent with the help of ultrasonication or even by gentle hand shaking [28].

MXene is a thermodynamically stable nanomaterial in the category of three-component ceramics and usually has dual behaviors of metallic (such as flexibility and high toughness) and ceramic properties (such as high elasticity, high abrasion resistance, low density, and good corrosion resistance). Unlike other hydrophobic nanomaterials, MXene nano-flakes can be easily used in different media without the need for any intermediate material in that environment [24]. The unique combination of hydrophilicity and conductive properties has made MXene nanoflakes a promising candidate for electrical and electronic applications; the conductivity and high concentration of surface functional groups make them particularly promising to be printed on a variety of substrates. Having a high viscosity of about 1–20 mPa s^−1^ and suitable surface tension is required for most printing purposes; further, the amount of surface tension must also correspond to the surface energy and the texture of the substrate to achieve a proper wetting condition. MXenes usually have a zeta potential of about -80 to -30 mV, leading to colloidal stability in water or other polar solvents [30]. 

Although extensive research efforts have been conducted to figure out different physical or chemical properties, rare studies have been reported about the rheological characteristics as well as the process printing parameters of the 2D MXene sheets. In this work, particular attention is paid to the inks based on MXene nanosheets. In terms of the layout of the present review, the formulation of MXene inks is briefly elaborated first, then the properties derived by both theoretical efforts and experimentations are presented and discussed in detail, followed by their applications in many different fields. Finally, the current research pitfalls, challenges and future opportunities will be provided at the end. 

## 2. Formulation of MXene Inks

### 2.1. Turning MAX into MXene

MXene is generally produced from a MAX phase through an acid etching, and then it is delaminated, intercalated and finally exfoliated by mechanical agitation like simple hand shaking or ultrasonication [28,31]. In an effort made by the Gogotsi group [24] to synthesize the first MXene (i.e., Ti_3_C_2_), Ti_3_AIC_2_ powder was immersed in a hydrofluoric acid (HF) of 50% concentration for 24 h; the resulting suspension was then washed in a distilled water, and then the compound was centrifuged to prepare a precipitate. It is worth noting that Al-Ti bonding to Ti-C is easily detachable [32]; a chemical etching with HF acid could remove Al from the original MAX material through the following reactions:

Ti_3_AIC_2_ + 3HF → AlF_3_ + 1.5H_2_ + Ti_3_C_2_ (1)

Ti_3_C_2_ + 2H_2_O → Ti_3_C_2_(OH)_2_ + H_2_ (2)

Ti_3_C_2_ + 2HF →Ti_3_C_2_F_2_ + H_2_ (3)

Reactions 2 and 3 indicate the formation of surface functional groups, i.e., OH and F, on Ti_3_C_2_T_x_ [33,34]. Selective etching by HF has a few major drawbacks, like etchant-induce defects, based on which the use of alternative mild etchants is almost always recommended to etch off with safer and faster strategies. Further, the quality of the final MXene strongly depends on the raw materials as well as the processing conditions. For instance, the M-A bond energy determines the time and concentration required for the hydrofluoric acid [29]; reducing the particle size of the MAX phase by a simple ball milling process may greatly reduce the synthesis time with a milder etchant type. 

Ghidiu et al. [35] removed Al from the Ti_3_AIC_2_ MAX phase in one step using HCI acid and LiF salts to increase the efficiency, speed, and flexibility of the MXene production process wherein the hydrofluoric acid is in-situ produced locally; a certain amount of LiF is dissolved in HCl at a concentration of 6 M; the Ti_3_AIC_2_ powder was then slowly added to the solution with the temperature kept in 40 °C for 45 h. Upon cooling down to ambient temperature, the product was then washed, centrifuged several times, and then dried. Ti_3_C_2_T_x_ was obtained with superior hydrophilicity and excellent electrical conductivity. In an effort to prepare Ti_3_C_2_T_x_ by a top-down technique, Shen et al. [36] used a facile hydrothermal method through immersing Ti_3_AIC_2_ powder in NH_4_F solution and heating in a stainless steel autoclave at 100 °C for 24 h to produce the final MXene. Liu et al. [37] conducted a study on the effects of various fluoride salts such as LiF, NaF, KF, and NH_4_F in HCl in etching Ti_3_AlC_2_ and Ti_2_AlC. According to Halim et al. [38], when NH_4_HF_2_ is used as an etchant, the etching time increases as compared to HF; however, NH_4_HF_2_ is able to put NH_4_^+^ ions between the MXene layers without the need for another layering agent; further, sodium, potassium, magnesium, and aluminum ions among the MXene layers not only help ion intercalation and delamination but also increase the volumetric capacitance in supercapacitors as shown in Figure 1d.

Surface terminations usually play a key role in the final properties, especially when dealing with the MXene inks and their flow parameters. For example, the bare/pristine MXenes (M_n+1_X_n_) usually have quite different features than those terminated MXenes (M_n+1_X_n_T_x_) [31]; almost all MXenes produced by top-down methods have surface functional groups, and rare studies have focused on the development of pristine MXenes (without end groups) being highly demanded in special applications like printing electronic devices. 

### 2.2. Composition of Inks

Ink is typically defined as a liquid of pigments and dyes for writing and printing. The ink composition is usually regulated in a manner to have suitable rheological properties for the desired printing process. Inks based on 2D nanomaterials are usually designed to serve particular functions. Thus, the appearance, or the gloss and color, are not given much attention like graphical inks. Over the past years, interest in composite materials/hybrids, particularly those conductive, semi-conductive, and dielectric materials typically used as active pigments in inks, has been growing. Metal nanoparticles [39], dielectric and organic semiconductors [40,41], carbon allotropes [42,43], and two-dimensional materials [12] are widely used materials having a great potential to tune the ink properties. As discussed below, the most common ink formulations are pigments, adhesives, solvents, and additives.

1. Adhesives mainly consist of polymer nanocomposites to act as films to link the pigments and thereby adhere to a given substrate. There are various polymeric resins, including acrylics, alkyds, cellulose derivatives, and plastic resins. The adhesives used in the ink formulation may affect, to some extent, the printing properties, such as ink gloss, air resistance, chemical resistance, etc. [44];
2. As volatile active ingredients, solvents maintain the ink in a liquid state during printing until a bottom layer is applied. Solvent type and amount vastly influence the final features, among which viscosity and rheology rank highly. A wide variety of organic and inorganic solvents can be utilized as the solvent. Finally, the choice of solvent type may considerably depend on printing technology, substrate, and application [44];
3. Inks are formulated with additives to change their properties; however, the additives are often filled with inks in very small amounts due to their substantial influence. In addition to surfactants being used to make pigments wettable, there are also emulsifiers that facilitate combining pigments with other substances. To make water-based inks more water-resistant, alkalis can be added to create a slight change in pH. Silicone-based additives are typically used to prevent bubbles during printing, making the printed film more resistant to moisture. Those ink containing 2D materials are quite similar to those graphical ink systems [45]. Reduced graphene oxide (rGO) [46], molybdenum disulfide [47], hexagonal boron nitride [48], black phosphorus [49], etc., have already been employed to produce advanced nano-inks. However, the formulations based on 2D materials are different from traditional systems owing to their special nature. For example, many two-dimensional materials are pre-dispersed in a liquid form, or they may require exfoliation from their bulk condition, while most materials are powdered in traditional systems [50,51]. In the next section, the preparation of inks filled with 2D additives is elaborated on and discussed further.

### 2.3. 2D Inks Preparation

Unlike remarkable scientific achievements in preparing inks with 2D materials, they have not yet been widely utilized in industrial applications. The formula of conductive inks well suited with diverse patterning strategies is crucial for fabricating low-cost, flexible electronics and electrochemical devices [52,53]. Owing to high surface charge and hydrophilicity, MXene inks could simply be applied on a variety of substrates using different processes like writing, printing, stamping, and painting; however, MXene inks up to now are seldom composed of solely delaminated-MXene flakes (i.e., the frequent presence of bulky structures in the deposited flakes), and hence the films made from the inks do not usually exhibit laterally stacked morphologies. The uneven stacking of MXene nanosheets may lead to a reduction in conductivity, therefore restricting the potential applications in electrical circuits or electrodes for energy storage devices. It is believed that formulating MXene ink with ideal elastic properties, permitting the 2D MXene flakes to be deposited uniformly and laterally, is urgently needed at the moment.

Since preparation methods usually consist of multi-stage processing and complex instruments [5,54], developing simple and cost-effective strategies to simulate conductive materials on various substrates or platforms seems to be highly demanded. Ranging from electrochemical capacitors to electromagnetic shielding, MXenes have shown promise in many fields [55,56]. By imparting hydrophilic surface functional groups to facilitate the synthesis of dispersion solvent [57,58], MXenes can be transformed into independent films through different processes like vacuum filtration, thin films on rigid substrates and flexible films using solution spray/spin coating [59,60], screen printing on paper or metal foil with a squeegee [61].

**Figure 2:**
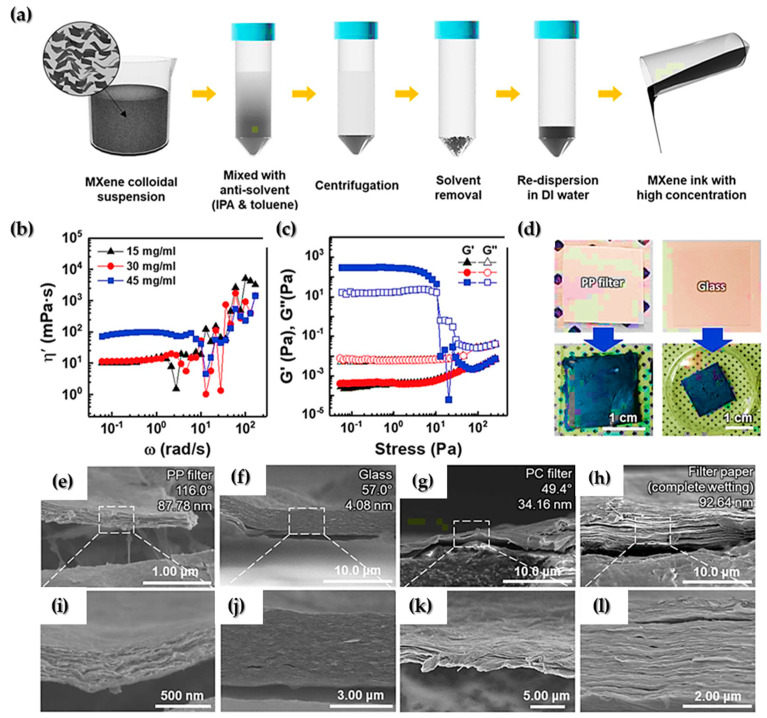
(**a**) Schematic illustration of the fabrication process of MXene ink. (**b**) Dynamic shear viscosity (ɳ’) curves of MXene inks at various concentrations and (**c**) the storage (*G′*) and loss (*G″*) moduli curves of MXene inks as a function of shear stress at different flake concentrations. (**d**) Painted MXene ink on various substrates using a brush. SEM cross−sectional images of painted MXene ink on the (**e**,**i**) PP filter, (**f**,**j**) glass, (**g**,**k**) PC filter, and (**h**,**l**) filter paper. Detailed information (i.e., contact angle and root−mean−square surface roughness) on the bare substrates is included on the upper right in (**e**–**h**), reprinted with permission from [62].

Seok et al. [62] used a vacuum filtering approach followed by an optional equalization procedure to produce a high-purity MAX phase precursor and MXene, wherein a high conductivity and enhanced EMI shielding behavior could be obtained without using any additives. Based on their rheological examinations, it was also found that MXene ink of lower than 45 mg mL^−1^ concentration may be printed on a variety of surfaces with ease, as demonstrated in Figure 2. In other words, the inks with less than 45 mg mL^−1^ concentration were considered suitable for liquid-like inks due to their stable rheological characteristics [62].

**Figure 3:**
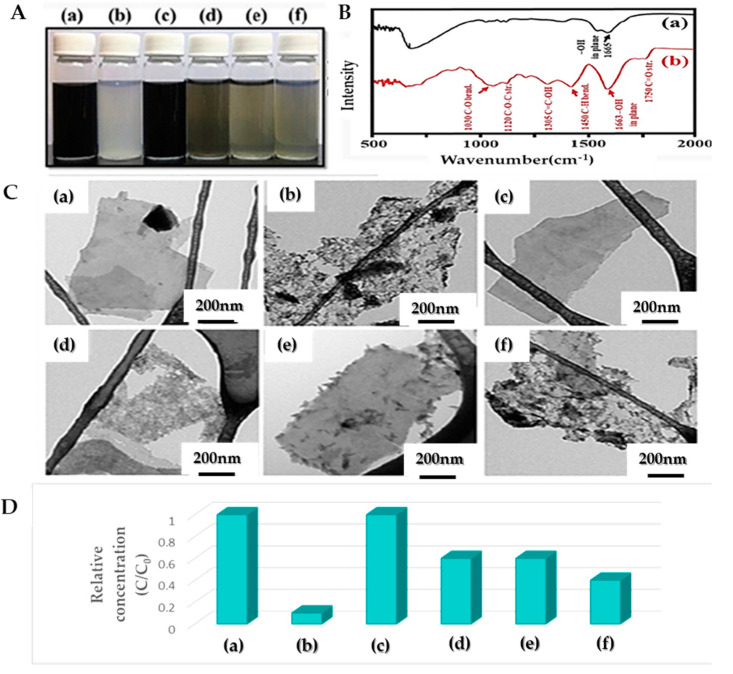
(**A**) Photographs, (**B**) FT-IR spectra, (**C**) TEM images, and (**D**) relative concentration (C/C0), (**a**) p-MXene-fresh, (**b**) p-MXene-80, (**c**) SA-MXene-80, (**d**) SC-MXene-80, (**e**) SOMXene-80, and (**f**) SP-MXene-80 suspension with the concentration 0.2 mg mL^−1^ in terms of Ti_3_C_2_T_x_ MXene. C0 and C in (**B**) represent the concentration of fresh and stored MXenes, respectively, reprinted with permission from [63].

As shown in Figure 3, to improve the dispersion and stability of the colloidal MXene suspension, Wu et al. [63] prepared MXenes by the selective etching of Ti_3_C_2_T_x_ and then tested four different ligands during the synthesis process, namely (i) sodium ascorbate (SA), (ii) sodium oxalate (SO), (iii) sodium citrate (SC), and (iv) sodium phosphate (SP) wherein it was revealed that ascorbic acid ions could greatly improve the dispersibility, oxidation resistance and that the distance between MXene layers was greatly increased being enhancing the charge/discharge capacity and specific capacitance characteristics. It seems the ascorbic acid may interact with the sub-coordinated Ti atoms of MXene through hydrogen bonds and coordination bonds on the edges of MXene. It was also found that SA-MXene has a higher cycle stability and specific capacity compared to other MXene samples [63].

Like the abovementioned research studies, the majority of studies conducted to date have dispersed MXene nanosheets in aqueous solvents; however, apart from the unique surface hydrophilicity, aqueous solvents present a number of critical issues such as degradation, long-term instability, as well as the increased incompatibility with local polymers in water [64]. One of the most pressing issues in contemporary research has been MXene dispersion in organic solvents. For instance, the tuned microenvironment method (TMM) was used by Zhang et al. [65] to disperse high concentrations of MXene in organic solvents wherein the MXene dispersion is accomplished even without the use of ultrasonic during the process; further, it was found that the insertion of TBA^+^, adjusting hydrophobicity through surface terminations and functionalizations are the key factors to achieve high-concentration MXene dispersion. Finally, since the size of MXene obtained by TMM was relatively larger than those dispersed in water, it is believed that the MXenes dispersed in organic solvents have higher oxidation resistance [65]. Carey et al. [66] could exfoliate and disperse MXene in non-polar solvents exhibiting high colloidal and oxidation stability; they transformed the lithium cations between the MXene layers by etching to dibenzyl methyl ammonium chloride to create an organophile to evaluate the dispersion and stability of MXene in non-polar liquids. In a non-polar, oxidation-free solvent, an extremely stable colloidal suspension of MXene could be generated and used for industrial settings. Wang et al. [67] suggested an easy method for converting MXene to a novel family of homogenous, solvent-free liquids in another work. Covalent bonding and surface engineering are the two foundations presented in this method. MXene-free liquid has antioxidant stability for 540 days and also has a macroscopic behavior at ambient temperature. Gas adsorption, photoluminescence, composite structures, and magnetic fluids all utilize this novel family of solvent-free liquids. It is believed the preparation of MXene ink still is in its infancy stage, and hence a lot of effort is still demanded to further improve the field. 

## 3. Properties of MXene Inks

### 3.1. Chemical Stability

As discussed earlier, MXene shows excellent dispersibility in various solvents, e.g., water [57,58,68]. Reports show that water-soluble oxygen has a detrimental effect on the chemical degradation of layered flakes, with the high aspect ratio affecting the structural decomposition and the reduced shelf life of MXene inks. In the presence of TiO_2_ on the Ti_3_C_2_T_x_ surface, for example, phase change and structural deformation occur when MXene is exposed to light or high temperatures [57]. Oxidation usually begins at the edges and then progresses to basal surfaces. While it may occur in all environments, those smaller flakes oxidize quicker than the large MXenes; meanwhile, The rate/speed of oxidation depends on the environment as it is maximum in liquids and minimum in solids [69]; therefore, it seems the elimination of the dissolved oxygen in water by saturating it with nitrogen or argon could be an effective way to suppress the oxidation rate. According to Zhang et al. [70], oxidation is very fast in air, and the MXene suspension is degraded by 1.2% after 25 days under argon gas protection. The possible oxidation mechanism is explained as follows [68].

Ti_3_C_2_O_2_ + 4H_2_O = 3TiO_2_ + 2C + 4H_2_ (4)

To avoid oxidation, MXene-based inks are usually stored in Ar-sealed vials for long-term storage; it is also recommended to store them at low temperatures (T < 4 °C) without exposure to light [68] since long-term storage is still challenging. Storing dried MXene in a vacuum or in an organic solvent could also be an option, as it has been observed that MXene inks remained stable in these conditions for several months [57]. It is noted that when stored in dry conditions, restacking may occur, particularly at elaborated temperatures. Based on research conducted by Zhao et al. [71], it has been revealed that antioxidants such as sodium ascorbate could improve oxidation resistance even in the presence of water and oxygen; sodium ascorbate binds to the positively charged edges of the MXene, resisting against oxidation deterioration. The benefit of antioxidants is that they usually do not change the chemical or colloidal stability or electrical conductivity; for example, in the case of Ti_3_C_2_T_x_, after 21 days, the properties were still well preserved. As an alternative strategy, polyionic salts such as polyphosphates or polysilicates can be used against oxidation owing to a few advantages such as easy washing and being cost-effective; they make the scalable production and long-term storage of MXene inks possible [72]. By developing a hydrogen annealing approach to enhance the oxidation resistance of bare MXene, Lee et al. [73] showed that the hydrogen treatment (i) could considerably improve the oxidation stability of MXene films in harsh conditions, i.e., at high temperatures and 100% relative humidity, (ii) hydrogen annealing could also restore the electrical conductivity of the previously oxidized films offering a great potential of MXene in real industrial applications.

### 3.2. Surface Properties

Based on preliminary studies [24], it has been predicted that terminated MXenes have negative formation energy with thermodynamic stability. Synthetic Ti_3_C_2_T_x_ usually have -OH, -O, or -F surface terminations [74]:

The concentration of functional groups depends on the etching conditions and pH [75,76]. Chemical etching can significantly alter the composition of Tx, and there is still no clear evidence of the identity and coordination of these surface-terminating groups; it is, therefore, important and interesting to study the coordination of T_x_ [77]. If high-concentration HF is used, more fluorine functional groups and fewer oxygen functional groups are formed; these surface terminations may lead to a hydrophilic surface with a negative zeta potential [75]. MXenes can be chemically modified to control their surface properties and to improve their stability by chemical modification [21], as the surface differences may result in novel chemical, mechanical, optical, and electronic properties [78]. Due to the unique surface properties, it is possible to use a stable suspension of MXene (e.g., Ti_3_C_2_T_x_) for the printing processes without additives such as surfactants and/or surface modifiers [79].

After the delamination process, MXene flakes usually have a lateral particle size of a few nano- to micro-scale range which can then be uniformed by centrifugation [80,81,82]. Surface properties highly depend on the flake size [83]; for example, according to Kylie et al. [84], the electrochemical properties of Ti_3_C_2_T_x_ can be improved by controlling the particle size and distribution; smaller flakes usually have less conductivity than large ones attributing to the contact resistance between the flakes due to the defect appeared on the edges.

### 3.3. Rheological Properties

#### 3.3.1. Aqueous Single-Layer MXene

Although the first member of the MXene family was discovered about a decade ago, attention on the rheological aspects of this 2D material has only come in the past few years [85]. According to Web of Science, the number of publications on MXene inks is 2, 7, 13, and 23, respectively, in 2017, 2018, 2019, and 2020 while the number of publications on MXene rheology is only 0, 2, 6, and 7, respectively [86]. According to Akuzum et al. [87], aqueous MXene has a viscoelastic nature with shear thinning behavior wherein the viscosity decreased by rotation speed while increased by the addition of higher MXene concentration; a zero-shear viscosity of 5 mPa s at 0.18 mg mL^−1^ was measured. Being an effective parameter in assessing rheological behavior, tan δ is calculated by the following equation:

(5) $$\tan\delta =\frac{G^{\prime \prime }}{G^{\prime }}$$

where δ is the phase difference between the applied stress (σ) and the corresponding strain (ε), and *G*″ and *G*′, respectively, represent loss modulus (viscous behavior) and elastic modulus (energy storage behavior). To further read about the basics of viscoelasticity, refer to [88]. It was found that for a low weight fraction of aqueous Ti_3_C_2_T*_x_* (< 0.90 mg mL^−1^), the behavior at low frequencies is mostly elastic with dominant *G*′ values; however, colloidal Ti_3_C_2_T*_x_* may exhibit a viscous-like behavior with dominant *G*″ values in a wide range of frequencies. The critical volume fraction of MXene nanoflakes ($\varphi^{*}$) is given by the following relation indicating the strong influence of surface charges and electromagnetic permittivity [89].

(6) $$\varphi^{*}\propto \frac{h}{d}{\left(1+\frac{2}{d\kappa }\right)}^{-3}$$

where *h*/*d* is the aspect ratio, and *κ*^−1^ is the Debye screening length. 

**Figure 4:**
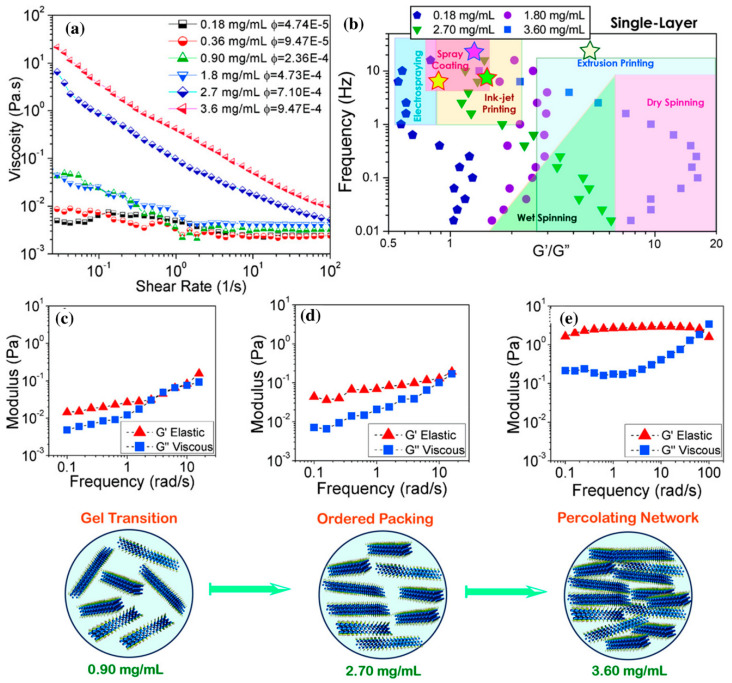
Viscoelastic response of 0.18–3.60 mg mL^−1^ (volume fraction, ϕ, 4.74 × 10^–5^–9.47 × 10^–4^) mono-layer Ti_3_C_2_T_x_ MXene in water. (**a**) Shear rate against viscosity (**b**) Frequency verses G′/G″ in different fabrication techniques. The stars show the approximate parameters used in MXene applications obtained from the literature. The viscoelastic behavior of MXene suspensions having (**c**) 0.9 mg mL^−1^, (**d**) 2.7 mg mL^−1^, and (**e**) 3.6 mg mL^−1^ MXene loadings under 0.1% strain amplitude, reprinted with permission from [87].

According to Akuzum et al. [87], *G′* is frequency dependent in higher weight fraction of MXenes (i.e., *G′* ≈ 2 Pa at 3.60 mg mL^−1^), being a suitable condition for those processes with weak gel-like wet spinning (Figure 4). Depending on weight fraction, viscosity, and elastic and loss modulus, Ti_3_C_2_T*_x_* can be used in a variety of techniques, such as spray coating, wet spinning, and inkjet printing, among others. 

It was seen that mono-layer Ti_3_C_2_T*_x_* could interact with each other even at a long distance via electrostatic forces. Single-layer MXenes usually have higher ζ-potentials than those multi-layers. For instance, it was seen that the rheological behavior might change at a relatively low weight fraction of 0.9 mg mL^−1^ Ti_3_C_2_T*_x_,* wherein the percolation threshold is reached with a gel transition occurring at or above the aforesaid concentration. 

Regarding the fluidity of a given ink, both static and dynamic yield stresses should be designed with care; the former represents a value needed for ink to initiate flowing and exist from the printing nozzle, while the latter is required to maintain the fluidity conditions; as a result, the ink should experience a stress of lower than the dynamic stress upon the ink adhesion to a given substrate.

Robot dispensing needs ink with the ability to withstand yield stress, surface tension and the weight of the next layers of printed ink, avoiding any shape distortion or unwanted strain during or after the printing process. To this end, *G′* must be greater than the sum of the applied forces, including weight and stresses; further, the amount of required yield stress is dependent on how quickly a transition between linear and non-linear viscoelastic behaviors occurs. Flow transition index (FTI), being the ratio of the oscillatory yield stress to the oscillatory stress at which *G*′ = 0.9*G*′*_LVR_*, is an important criterion to estimate the required yield stress; the smaller FTI is usually desirable.

When the weight fraction of aqueous monolayer Ti_3_C_2_T_x_ is < 10 mg mL^−1^, the storage modulus and the yield stress value are not appropriate for extrusion printing. However, the aqueous ink with a higher concentration than the percolation threshold may form 3D structures by direct ink writing (DIW).

Using a low-waste layer-by-layer extrusion printing technique, Yang et al. [90] could produce 3D freestanding MXene architectures with an ideal rheological property to be used in microsupercapacitors wherein the energy and power densities of 24.4 μWh cm^−2^ and 0.64 mW cm^−2^ were respectively achieved at 4.3 mA cm^−2^; it was seen an increase in the weight fraction of Ti_3_C_2_T_x_ MXene might greatly increase the viscosity and elasticity of the given ink system exhibiting the shear thinning of four orders of magnitudes and a quick transition from low (0.01 s^−1^) to high (1000 s^−1^) shear rates when 50 mg mL^−1^ Ti_3_C_2_T*_x_* (with *G*′*_LVR_* of 36.5 kPa and oscillatory stress value of 206 Pa) is used being promising for the DIW. 

The effect of aspect ratio and flake size on the rheological behavior of Ti_3_C_2_T_x_ MXene flakes in different solvents was studied by Zhang et al. [91], wherein they see both inks with small and large size nanosheets exhibiting a nematic phase behavior; however, those inks prepared with small-size flakes should be much more concentrated than the inks with larger MXene sheets to reach the similar values needed for viscosity and tan δ; they also believe that there is a critical transition concentration value (C_t_) above which a nematic phase can appear. Finally, 6.3 mg mL^−1^ is a concentration below which liquid-like and above which solid-like behaviors are seen, indicating a sharp transition from the liquid-to-solid-like behavior at this weight fraction. The effect of flake size on the rheological features has later been investigated by Yang et al. [92], confirming the role of flake size in governing the viscoelasticity of an MXene ink system. 

In order to tune the rheological properties of MXene inks, other approaches, such as using super absorbent polymer (SAP) beads, have already been examined wherein Orangi et al. [93] achieved a concentration of 290 mg mL^−1^ Ti_3_C_2_T_x_ (ca. 28.9 wt.%) with a yield stress of 24 Pa, a flow index of 0.73, and the value of tanδ in a deemed desirable range for extrusion printing. Shen et al. [94] used vacuum filtration to obtain a 3.5 mg mL^−1^ Ti_3_C_2_T*_x_* ink with decreasing viscosity with similar exponent reported by Orangi et al. 365 Pa 16.3 kPa, and 14 were the values of oscillatory yield stress, small strain storage modulus, and FTI, respectively, which is suitable for DWI. Greeves et al. proposed the following equation with good agreement with the data published in literature to show the effects of flake size (Z), weight fraction (C), and shear rate (γ˙) on the viscosity (η) of Ti_3_C_2_T*_x_* MXene inks:

(7) $$\eta \approx 1.1{\left(\mathrm{ZC}\right)}^{1.5}\gamma {˙}^{-0.9}$$

The discrepancy observed between the results obtained by the above equation and those experimental values reported to date is probably owing to other parameters such as pH, flake size distribution, dispersion quality, defects concentration, the age of colloid, as well as Zeta potential values affecting the final viscosity. 

#### 3.3.2. Aqueous Multi-Layer MXene

As discussed earlier, inks filled with single-layer MXenes exhibit shear thinning properties as well as viscoelastic behaviors; however, it has been proved that inks with multi-layer Ti_3_C_2_T*_x_* MXene also have viscoelastic behavior but at much higher loadings than mono-layer MXene inks [87]; a viscosity of 1770 Pa s with shear thinning behavior has been observed at 70 wt.% Ti_3_C_2_T*_x_* MXene ink. While the loss modulus is greater in lower weight fractions, the value of elastic modulus gradually increases to reach the loss modulus value; then, both loss and elastic values increase by orders of magnitude toward the percolation and gel transition conditions. Like those inks with single-layer MXenes, the rheological behavior of the inks with multi-layer MXene flakes can also be tuned by, for example, the weight fraction. The advantage of multi-layer MXenes over those single-layer flakes is their higher elastic modulus being an asset for printing purposes. However, owing to higher ζ-potentials and much stronger long-distance interactions of mono-layer MXenes, they show viscoelastic behaviors even at low MXene concentrations and moderate strain rates. 

By using a screen-printing process to produce conductive paths and micro-supercapacitors, Abdolhosseinzadeh et al. [95] first combined single- and multi-layer Ti_3_C_2_T*_x_* as well as un-etched Ti_3_AlC_2_ MAX phase to produce an ink with relatively low-shear viscosity of 35 Pa s and an elastic modulus of ca. 370 Pa. Afterwards, they diluted the compound to reach an ink containing 22 wt.% solid additives with the viscosity and the storage modulus of one and two orders of magnitudes, respectively.

#### 3.3.3. Non-Aqueous Inks 

Apart from water-based inks, many other non-aqueous solvents have also been utilized to produce MXene-based inks, such as dimethyl sulfoxide (DMSO) [96,97]. 

Vural et al. [97] filled 2.25 mg mL^−1^ Ti_3_C_2_T*_x_* to DMSO and observed the resultant ink exhibited a Newtonian rheological property having 3.1 mPa s viscosity in a wide range of shear rates of 2–200 s^−1^ wherein the surface tension was 51.5 mN m^−1^ and nozzle aperture 120 μm; although the addition of 0.95 mg mL^−1^ TR42 protein to the ink changed the inverse Ohnesorge number from 27.48 to 21.3, the ink could successfully be printed on a variety of substrate to produce flexible electrodes having excellent electrical conductivities. Zhang et al. not only examined ca. 12 mg mL^−1^ Ti_3_C_2_T*_x_* in DMSO ink, but they also studied other inks based on water, N-methyl-2-pyrrolidone (NMP), and ethanol wherein viscoelastic behavior was detected in all inks; they also found the values of inverse Ohnesorge number to be much lower for Ti_3_C_2_T*_x_* than the ones reported by Vural et al., indicating superiority of MXene over TR42 protein. The printed lines made by Zhang et al. [96] exhibited significantly high electrical conductivities of up to 2770 S cm^−1^.

**Figure 5:**
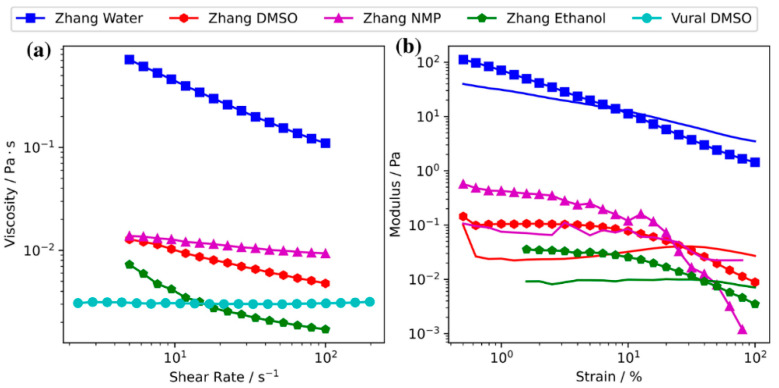
Comparison of rheological behavior of Ti_3_C_2_T*_x_* MXene-based inks reported by Zhang et al. and Vural et al. (**a**) Viscosity vs. shear rate. (**b**) Loss and elastic moduli vs. oscillatory strain amplitude (*G*″ without markers and *G*′ with markers). The amounts of Ti_3_C_2_T*_x_* MXene in water, ethanol and NMP are respectively 36, 0.7, and 12 mg mL^−1^. 12 mg mL^−1^ and 2.25 mg mL^−1^ are the Ti_3_C_2_T*_x_* concentrations in DMSO, respectively, used by Zhang et al. and Vural et al.

As seen in Figure 5, the rheological properties of different MXene-based inks are similar in terms of viscosity and shear rate behaviors. The MXene-in-water inks seem to have greater viscosity and shear rate values, probably owing to the higher viscosity of shear rate values of pure water than pure DMSO, NMP, and ethanol.

Apart from the abovementioned solvents, the rheology of MXene can also be studied when added to polymers or other hybrid materials. The number of studies in this area is few, and only a few sporadic papers have studied the rheology of melt polymers filled by MXenes [98]. Gao et al. dealt with the rheological properties of Ti_3_C_2_T*_x_* MXene/thermoplastic polyurethane nanocomposites at 200 °C; they dispersed 0–1.0 wt% Ti_3_C_2_T*_x_* nanosheets in thermoplastic polyurethane (TPU) wherein they observed both elastic and loss moduli increased with the frequency and that the highest viscosity was measured in the sample with the highest MXene loadings. 

It has been found that chemical modification or hybridization can be considered a rheology tuner. Yu et al. used nitrogen-doped Ti_3_C_2_T*_x_* (N-Ti_3_C_2_T*_x_*), carbon black, and a polymer in water to produce ink for screen printing; they also used N-Ti_3_C_2_T*_x_* hybridized with CNTs, activated carbon and graphene oxide in water to produce DIW ink. The former ink had a percolation threshold at about ca. 28 wt%, with no significant changes in *G*′ and *G*′′ above the mentioned threshold, indicating gel stability at oscillating frequencies. Further, the N-Ti_3_C_2_T*_x_*/C ink with 34 wt.% solid loadings exhibited a significant viscosity at relatively low shear rates with subsequent high-resolution printed electrodes. The elastic modulus and yield stress value of the N-Ti_3_C_2_T*_x_*/AC/CNT/GO ink with 15 wt.% solid loadings were respectively reported as 2 and 3.5 times higher than the ink without MXene, with 11 wt.% AC/CNT/GO filler.

**Figure 6:**
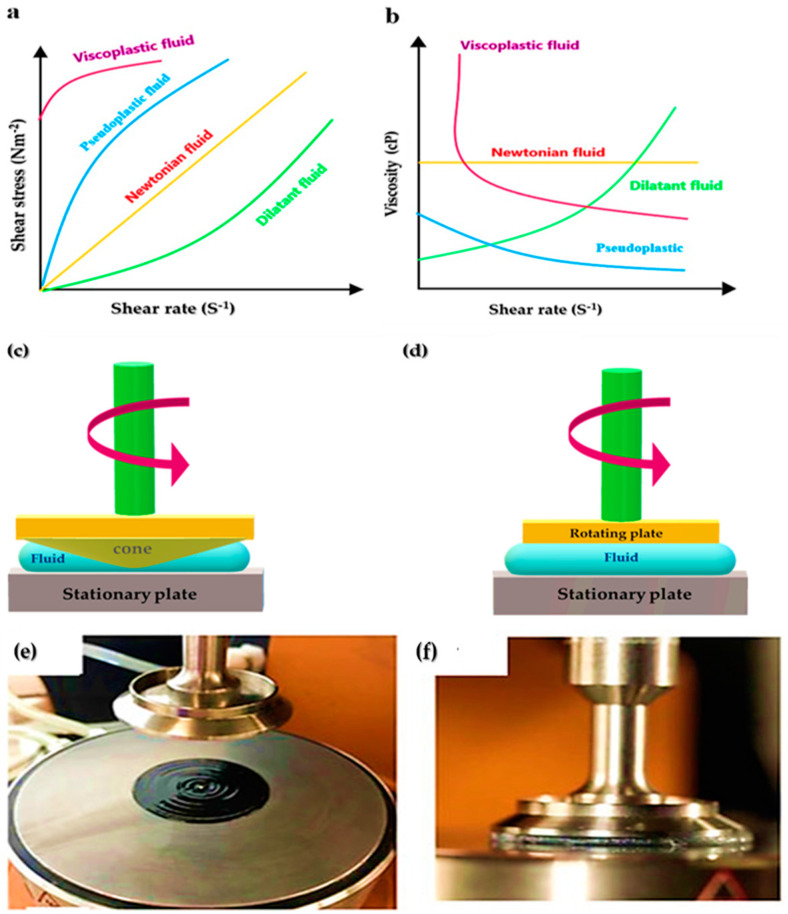
(**a**) Shear stress and shear strain flow curves for different fluids and (**b**) viscosity profiles for different fluid systems (**c**) Schematics (not to scale) of CTP and (**d**) PTP viscometers. Photograph of a PTP setup preparing for measurement with (**e**) plate retracted and ink loaded and (**f**) plate down on an ink sample, reprinted with permission from [44].

Although a number of parameters, such as drying time, dot gain, and water pickup, are important, ink rheology and viscosity are the two main parameters that govern printability. Rheology can be defined as how ink flows through the various stages of printing; further, the difference between the term rheology and viscosity is significant. Rheology is the study of fluid over time and the shear and stress gaps, while viscosity is the resistance to liquid flow at a given time. Where its rheology governs how these external forces affect the ink is itself invariant. Figure 6a,b graphically shows the relationship between shear stress and shear strain in a fluid [99,100]. In a Newtonian flow of an ideal fluid, a linear relationship between shear stress and shear strain is observed. The viscosity remains constant for different shear rates. It should be noted that no liquid is completely Newtonian. This issue has been fully discussed elsewhere [44].

There are many ways to describe rheology and measure it for inks. Plate-to-plate (PTP) and cone-to-plate (CTP) systems are generally more expensive than conventional methods. Still, beneficial information about the rheological performance of non-Newtonian fluids can be obtained from the results. The principle behind CTP and PTP viscometers is shown in Figure 6c,d [44].

The physical behavior of MXene under applied stresses determines the rheology of MXene ink and is an influential factor in the final print, its quality and performance. Both pseudoplastic and thixotropic properties are descriptive of shear thinners. Shear-thinning fluids are efficient for various printing techniques such as extrusion, screen printing, and spray coating. This type of liquid, when a force is applied, passes through the printer nozzle and after leaving the nozzle, it has a high viscosity, which helps maintain the printing pattern [75,101].

**Figure 7:**
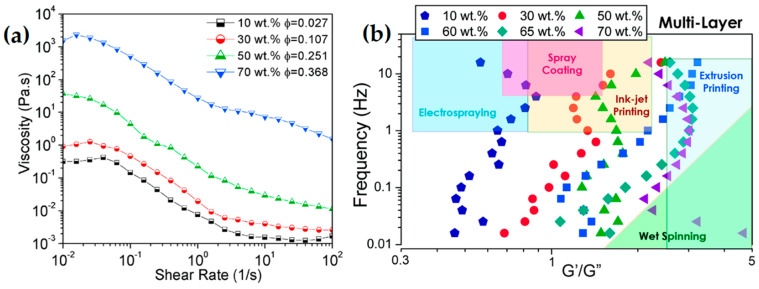
(**a**) Measured viscosity versus the shear rate of multi−layer MXene flake suspension (**b**) Frequency dependence of the ratio of the G′ elastic modulus to G″ viscous modulus for multi-layer Ti_3_C_2_T_x_ MXene flakes dispersed in water, reprinted with permission from [87].

Shear thinning behavior has been observed for MXene inks based on both mono-layered and multi-layered MXenes. The ratio of elastic modulus (G′) to viscous modulus (G″) is another important parameter that determines the rheology of dispersion. Of course, the rheological requirements for different printing methods are different. For example, spray coating requires a high processing rate with a high viscosity modulus; on the other hand, extrusion printing requires a high elastic modulus to maintain the desired print pattern. According to Figure 4a,b and Figure 7, it can be seen that single and multilayered MXenes have shown different behaviors [90].

To match the surface tension between the ink and the substrate, high-quality dispersion of MXene (e.g., Ti_3_C_2_T_x_) in different solvents is required. Among the polar solvents suitable for this part, the following can be mentioned [57,65,102]:

1. N-dimethyl formamide (DMF);
2. N-methyl-2-pyrrolidone (NMP);
3. Dimethyl sulfoxide (DMSO);
4. Propylene carbonate (PC);
5. Ethanol.

It should be noted that flakes dispersed in organic polar solvents have better resistance to oxidation than water. The concentration of dispersed Ti_3_C_2_T_x_ and the viscosity of the solvents mentioned above have a linear relationship indicating that there is a fluid flow of ink when these solvents are used for various printing techniques [57].

A wide range of rheological properties and different concentrations make it possible to print MXene with different techniques. Due to this reason, MXene inks have been very popular among researchers for various applications, some of which are briefly mentioned in the next section.

## 4. Printing Methods

Although printing has made great strides to date, functional printing and ink development still remain in their infancy. Owing to the difficulty of developing high-performance additive-free 2D inks, great efforts are needed. Among nanomaterials, MXenes have special capabilities offering extraordinary production possibilities [103]. To make MXene films with a thickness of nanometers and micrometers for practical applications, one needs to be able to deposit patterned solid-state dispersions of MXene [75]. There have been several reported methods of printing/coating for achieving this goal [59,96,104,105,106]. Various printing and coating techniques have differences in fluidic properties, resolution, and scalability [75,103]. 

### 4.1. Inkjet Printing

The technology of inkjet printing (IJP) is a digital, non-contact method that is extensively used in research as well as industrial application [103]. Considering that a pattern is provided to the printer as a digital file (which can be modified easily) and the ink consumption is very low (1–2 mL), this is one of the best techniques for parameter optimization and fast prototyping [107]. There are two main types of inkjet printing based on the droplet generation mechanism, i.e., continuous inkjet printing (C-IJP) and drop-on-demand printing (DOD-IJP); C-IJP is mainly used in industry, while DOD-IJP is more appropriate for scientific research in the lab [108].

One of the major challenges in inkjet printing is producing printable inks with suitable physical fluid properties. A good indicator for fluidic properties and ink printability is the inverse Ohnesorge number Z, which is defined as [75]:

(8) $$Z=\frac{\gamma^{1/2}}{\eta }$$

where α is the printer nozzle diameter, η is fluidic density, γ is the surface tension, and η is the fluidic viscosity. Jang et al. recommend an optimal Z range of 4–14 for inkjet-printable ink. Based on the rheological properties of MXene inks, the Z value of (~2.6) for ethanol ink is slightly higher than for DMSO, with a Z value of (~2.5) and NMP ink with a Z of (~2.2). Optimal Z-values for MXene organic inks are (1 ≤ Z ≤ 14) [75].

Wearable electrical biosensors with a great degree of pattern flexibility could be made using the process of inkjet printing, which is a simple and basic route. Inkjet printing with digital patterns eliminates the need for a mask, simplifying the fabrication process. This reduces the amount of material wasted in the process of creating the desired pattern. Owing to their solution-processability, surface chemistry, superior conductivity, and biocompatibility, MXenes sheets have attracted great attention for printing different films such as those used in epidermal applications, out of which only two investigations so far have used MXenes in skin electronics, one to estimate skin bending and another to detect hydrogen peroxide in perspiration [109].

**Figure 8:**
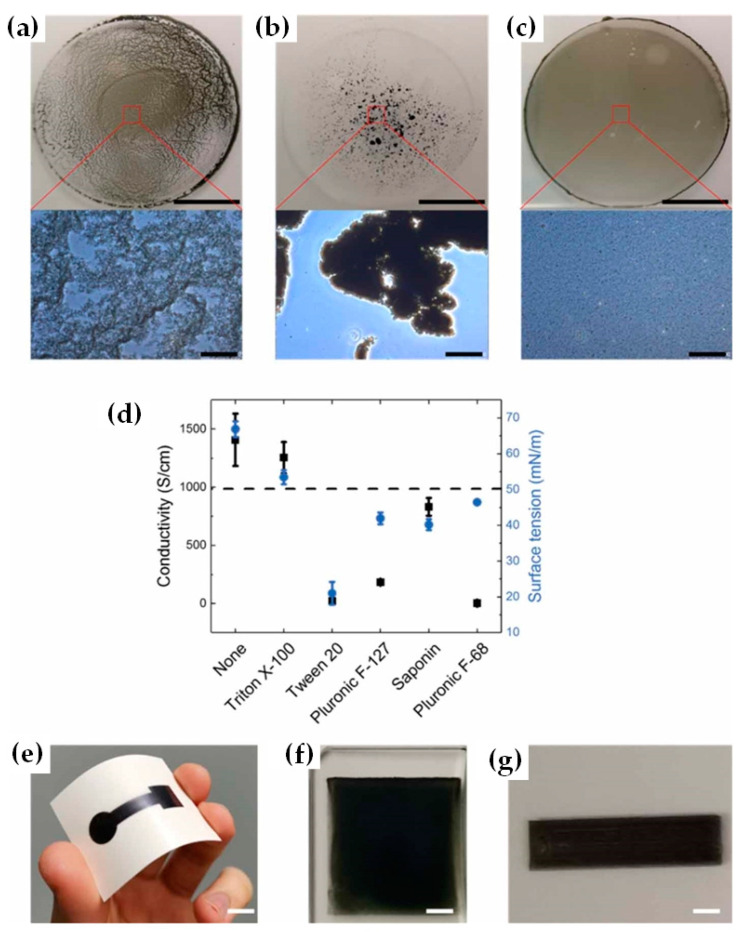
Optical photographs of MXene solutions comprising (**a**) an anionic and (**b**) a cationic surfactant, both creating heterogeneous suspensions. (**c**) MXene solution with a nonionic surfactant creates a homogeneous suspension. The bottom images are microscope images of the same suspensions. (**d**) The effect of surfactants on the surface tension of MXene inks and the conductivity of the resulting films (n = 3). Digital photographs of MXene films printed on various substrates: (**e**) tattoo paper, (**f**) glass and (**g**) a PEDOT:PSS coated glass substrate. Scale bars are 5 mm (**a**–**c** top), 100 µm (**a**–**c** bottom), 1 cm (**e**), and 2mm (**f**,**g**), reprinted with permission from [110].

As a biosensing platform, Saleh et al. [110] created an aqueous MXene ink and inkjet-printed MXene sheets on flexible, conductive polymer substrates, as demonstrated in Figure 8. As part of MXene dispersion, a nonionic surfactant saponin is added to the electrodes that can be inkjet printed with water-based MXenes and attached to the skin as a biosensor. An ion-selective layer on printed MXene surfaces can be used to create a Na^+^ sensor in a sweat-like medium. In addition to a good signal-to-noise ratio, these electrodes are durable, having a shelf life of 50 days in ambient conditions. In addition to paper, glass, and PEDOT: PSS film, the MXene/saponin formulation is printable on a wide range of substrates that have varied mechanical and surface properties. Sticky tapes were used to test for the adhesiveness of the MXene films on these substrates. Paper was less adherent to MXene than it was to glass. This paper shows that MXene printed films may be used for multifunctional biosensors, as Na^+^ in artificial sweat can be detected when combined with a sodium (Na^+^) ion-selective membrane (Na^+^ membrane). Interferon Gamma (a proinflammatory cytokine protein) causes antibodies on films to generate an electrical signal. As a result, by mounting IFN antibodies on the surface of the films, they might be employed as cytokine protein sensors. They’ve created a platform that shows how printed MXenes could be employed in a variety of sensing applications.

Vural et al. [97] developed 2D MXene inkjet printing, utilizing protein-based binders that can build sequence-controlled assemblies with hydrogen bonding 2D crystals. Cellulose paper, glass, polyethylene terephthalate (PET), and polymethylmethacrylate (PMMA) were all effectively printed with these inks, and they printed MXene patterns on PET films to create deformable light-emitting diode (LED) circuits and EMI shielding materials to demonstrate the potential of these inks in flexible electronics. This technique might be useful in the development of humidity-mediated memory systems and sensors, especially for microfluidic systems.

MXene inks have rarely been inkjet-printed on textiles, which demand inks with different characteristics than those required for nonporous surfaces [111]. Using a commercial printer, Uzun et al. [111] showed that these inks may be utilized to TIJ print different structures such as conductive patterns and interdigitated devices; they showed how controlled ejection of ink droplets from nozzles onto textile substrates like knit and woven textiles might be used to make wearable micro-supercapacitors (MSCs); this was the first time MXenes have been printed on textiles for textile-based wearable devices. On all surfaces, increasing the Ti_3_C_2_T_x_ flake size and concentration resulted in printed lines with reduced resistance. The inkjet-printed circuit on a cotton knit fabric produced with low concentration (9.4 mg mL^−1^) was found to have adequate conductivity to operate three LED lights after only one print pass. When six print passes were conducted, the electrical resistance of the lines ranging from 450 cm^−1^ (S-Ti_3_C_2_T_x_ ink at 22.4 cm^−1^) to 55 cm^−1^. Over several cycles, the devices showed outstanding cycling stability and generated reasonably high energy and power densities of 12.36 ± 4.46 Wh cm^−2^ and 0.16 ± 0.58 mW cm^−2^, respectively. In this way, the functioning of materials that may be printed for a range of textile-based energy harvesting, storage, and sensing applications was greatly improved [111].

Inkjet printing is a cutting-edge strategy for utilizing the benefits of 2D materials for printed optoelectronic devices with tiny small-footprints, integration, substrate/geometrics compatibility, scalability, and low cost. The selection of ink solutions is crucial. For ink formulation, high-viscosity solutions such as N-methyl-2-pyrrolidone (NMP) and dimethyl sulfoxide (DMSO) can be employed directly. To improve the printed film uniformity, a small amount of polymers or proteins can be added to the solutions that act as “binders”. Jiang et al. inkjet-printed MXene nanosheets in laser resonators with both fiber and free-space geometrics, achieving a wide range of spectral band ultrafast laser operations from near-infrared to mid-infrared, with pulse durations up to 100 femtoseconds [112].

Inkjet printing offers the greatest potential for rapidly creating and using novel materials. Wen et al. [109] reported an early example of inkjet-printed MXene transparent films. The key in this study is how to overcome the “coffee ring effect,” which complicates the inkjet printing process; it is extremely difficult to manufacture large-area transparent film electrodes due to the fact that the majority of the solute is deposited at the edges of the printed patterns. Furthermore, ink rheological properties and the solvent used in MXene ink affect printing performance. Films made with Ti_3_C_2_T_x_ can serve both as transparent conductors and capacitors. They may be used to build symmetric or asymmetric flexible and transparent supercapacitors to increase energy and power densities.

The extensive research done in inkjet printing has provided the technique with many benefits, such as the ability to print complex patterns, the use of different substrates with different properties, and high resolution. All of these point toward the ability to make micrometer-sized devices. However, in addition to all the advantages, some issues are of fundamental importance, especially the preparation of inks with appropriate rheological properties, fluidity, and appropriate physical and morphological characteristics [103].

### 4.2. Screen Printing

The screen printing method (SP) is a fast and efficient method of direct printing of 2D materials [103], and the ink is deposited via a mesh with some open (printing pattern) and blocked (non-printing) areas. Screen printing is basically divided into two methods: flat-bed and rotary [75]. In detail, the flat-bed approach involves pressing ink through a flat, patterned screen onto a substrate and repeating this process to build up layers, while in the rotary process, ink is pressed through an ink cylinder and perforated metal onto the substrate. MXene sediment printing illustrates the feasibility of making wearable smart electronics using waste-free ink formulation for screen printing.

**Figure 9:**
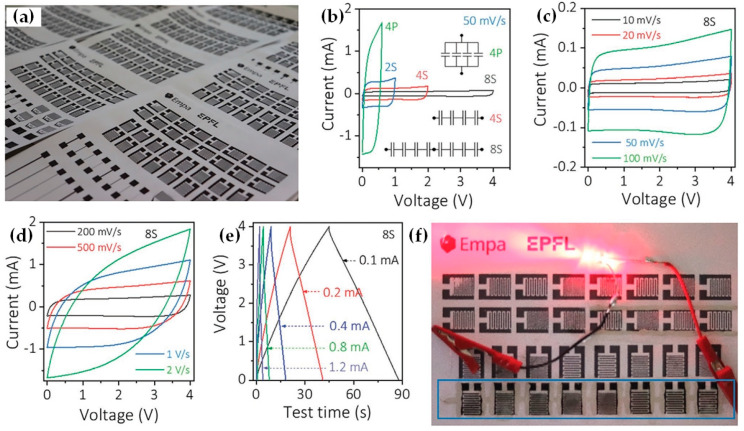
Scalable production of micro-supercapacitors based on MXene sediment inks. (**a**) Optical image of a screen-printed MXene-based microsupercapacitor, showing the great promise of scale-up production of high-performance MSCs. (**b**) CV profiles of different tandem devices at 50 mV s-1. (**c**,**d**) CVs of a tandem device with eight MSCs connected in series at different scan rates show a high-rate response. (**e**) GCD profiles of a tandem device with eight MSCs connected in series, indicating symmetric, linear curves at various currents. (**f**) Optical image of the tandem device to power an LED light, demonstrating the feasibility of the as-printed tandem device for practical applications, reprinted with permission from [95].

S.Abdolhosseinzadeh et al. [95] demonstrate scalable printing of diverse structures and patterns using additive-free MXene sediment inks with good spatial regularity and high resolution. With the help of patterns with different line and gap sizes, it is possible to create a conductive pattern, letters, and MSCs in a matter of seconds (Figure 9a). To provide energy and power solutions for unique applications, the all-printed MSCs may be linked fast and simply in series or parallel (Figure 9b). All tandem devices have perfect capacitive behavior and little IR drop (Figure 9c,d), making them the optimal approach. Figure 9d,e shows that the tandem device has a very low equivalent series resistance due to its rapid charge/discharge rate, and “yrashed” sediments from MXene may easily power a bright LED (Figure 9f), their high capacitance (158 mF cm^−2^) and energy density (1.64 watts cm^−2^) set them apart from other printed MSCs in terms of energy density and areal capacitance [95].

There are several advantages to the screen-printing method, such as controllable thickness, cost-effectiveness, and mass-production capabilities. Conversely, its major disadvantages are low print resolution, roughness, and thick ink used in this process [75]; all of them indicating that still a great effort should be made in this area.

### 4.3. 3D Printing

Using 3D printing technology, complex-shaped parts can be formed automatically from CAD data (computer-aided design) without any tooling [11]. Extrusion-based additive manufacturing (AM) is a process for fabricating complex structures utilized in a variety of industries, including energy storage. An ink (filament) of a colloidal or gel-type is extruded from a nozzle and layered on a surface to form 3D objects in this printing process [93]. A 3D object is created by layering materials upon each other in additive manufacturing [113]. Unlike previous 2D printing methods, 3D ink consists of polymers that are usually solid-liquid or powder. Comparable to other manual or molding processes, this automated fabrication has some benefits, such as computer-aided design capabilities, fast editing of CAD models and precise dimensioning [114]. A 3D printing process can be summarized as follows: (1) designing a CAD model, (2) conversion of the model to an STL file, (3) slicing the STL file to several 2D layered cross-sections, (4) 3D printing the prototypes, and then (5) post-processing [115].

In most cases, 3D-printed electrode materials are not electrocatalytic or appropriate for these purposes. Further modification of 3D-printed electrodes can be achieved using atomic layer deposition and electrodeposition. The only downside is that electrodeposition or ALD may not be possible for all materials since not all precursors are available. Electrodeposition and ALD currently cannot be used to deposit MXenes [116,117,118]. Using highly concentrated MXene ink for extrusion printing at room temperature, Orangi et al. [93] demonstrate that MSCs with a range of topologies and electrode thicknesses can be produced at a large scale. It is possible to print flexible MSCs on polymer and paper substrates using established printing methods. The aforesaid research describes printed solid-state devices exhibiting a very high capacitance area and excellent electrochemical performance.

Regarding 3D bio-printing, Rastin et al. [119] demonstrate that Ti_3_C_2_ MXene nanosheets can be used to build 3D bio-inks despite the fact that most of the current bio-inks have poor electrical conductivity. Studies have shown that hydrogels with electrical conductivity have improved signal transmission between cells. In most cases, these conductive bionics were developed by combining biopolymers with conductive polymers, but studies of 2D conductive materials were limited. It was found that HA/Alg biogels containing Ti_3_C_2_ nanosheets have outstanding printability and good resolution. The conductivity of the mentioned biocomposite ink could reach 5500± 85 μs cm^−1^ and 7200 ± 126 μs cm^−1^, respectively, when 1 mg mL^−1^ and 5 mg mL^−1^ of Ti_3_C_2_ were respectively added to the ink, surprisingly higher than those values obtained from the neat ink, being promising for many biomedical applications such as neural tissue engineering and the like [119].

**Figure 10:**
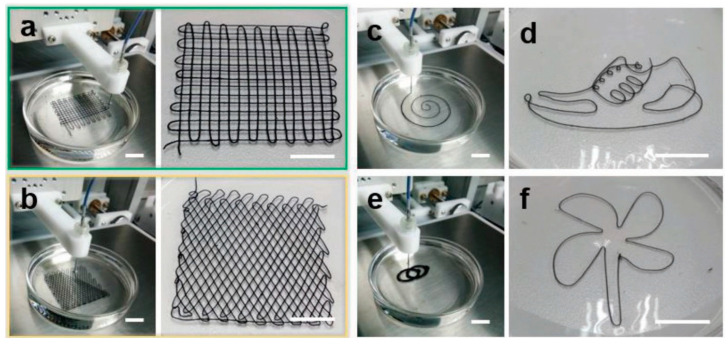
Optical images of the 3D-printed TOCNFs/Ti_3_C_2_ fabrics with (**a**) woodpile and (**b**) fishing net structures. (**c**–**f**) Programmable printed TOCNFs/Ti_3_C_2_ fibers with designed geometric structures, reprinted with permission from [120].

3D printing is also an optimal solution for integrating functional materials into multidimensional architectures. Cao et al. [120] successfully developed TOCNFs/Ti_3_C smart fibers and textiles with woodpile and fishing net structures with enhanced electrical, photonic and mechanical properties (Figure 10); this type of hybrid inks is promising in versatile applications such as smart textiles, electronic skins, wearable sensors, soft robotics, and human-machine interaction [120].

**Figure 11:**
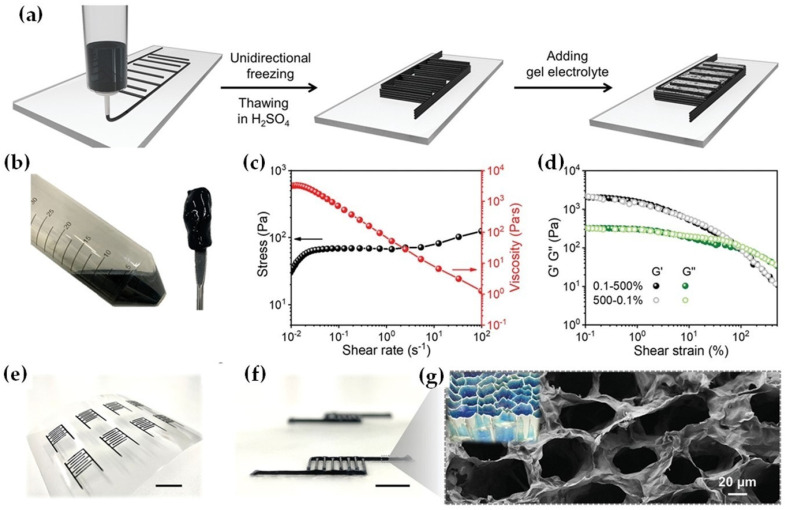
(**a**) The fabrication process of 3D-printing all-MXene MSC via MSES. (**b**) The digital photographs of MXene slurry. (**c**) Shear-thinning behavior of the MXene inks (stress and viscosity against different shear rates). (**d**) The oscillatory measurements (1 Hz) of the MXene ink, which sweeps from 0.1% to 500% and back to 0.1% strain. (**e**,**f**) Photographs of 3D-printed MXene MSC, scale bar is 1 cm. (**g**) The top-view SEM image of MXene hydrogel MSC, reprinted with permission from [121].

Huang et al. [121] used an in-situ ice template to produce MXene/CNT aerogel electrodes through a 3D printing approach. During the surfactant/MXene assembly process, shear stress is applied to align the flakes vertically. Three-dimensionally printed all-MXene micro-supercapacitors (MSCs) can provide an ultrahigh capacitance of 2.0 F cm^−2^ at 1.2 mAcm^−2^ and retain a record-high energy density (0.1 mwh cm^−2^ at 0.38 mW cm^−2^) [121]. 3D printing ink relies on extrusion-based technology to ensure smooth filament extrusion with good accuracy and shape retention. It is possible to create oriented microstructures even in high concentrations of MXene, and this 3D printing strategy may be applicable for additive manufacturing of MXene micro-supercapacitors as shown in Figure 11a, wherein MXene slurry forms a viscous, thixotropic ink with 3.3 × 10^3^ Pa s and without aggregates (Figure 11c). A scatter plot displaying the storage modulus and loss modulus of MXene ink as a function of shear strain is shown in Figure 11d. Plateaus of *G′* are orders of magnitude greater than those of *G*, which show primarily solid-like behavior. Extrusion will continue through micron-sized nozzles at relatively low pressures using this feature [121].

In a recent study on 3D printing MXene-based hybrid ink, Jambhulkar et al. [122] explained how a hybrid process is able to obtain nano-aligned and micropatterned MXene nanoflakes with hierarchical 2D morphologies. The developed 3D printing process included a surface topography design by a microcontinuous liquid interface production (μCLIP) as well as a direct nanoparticle-assembling through capillarity-driven DIW method wherein the final hierarchical product exhibited anisotropic conductivity values with a wide piezoresistive sensing range, and enhanced mechanical durability. Using this hybrid 3D printing technology, nanoparticles can be patterned and assembled for broad applications in a fast and scalable manner, indicating both manufacturing feasibility and device functionality [122].

In all, 3D printing has many advantages, including environmental friendliness, economic efficiency, controllable thickness and geometry, and flexibility of the printed pattern; however, it is limited by the materials that can be used for printing, being the most critical challenge of this method [75].

### 4.4. Stamping

There are several methods of non-pattern printing, including screen printing, spray coating, and stamping. Stamping is typically used to create effective deposition, but the adhesion between the ink, donor, and substrate requires to be precisely optimized. High printing speeds and scalability are the advantages of stamping, while the high cost of setting up and prototyping gravure rollers and plates, as well as the large volume requirements for inks, are the major disadvantages of this technique [75]. Stamping is a good option for producing flexible, scalable, and effective devices. Inks used for stamping should have characteristics such as high electrical conductivity, a long service life, excellent mechanical properties, good strength to withstand deformation, as well as excellent physical properties such as rheology, viscosity, surface tension, and adhesion [123,124].

This type of manufacturing method is divided into four categories depending on the use of a template: gravure, letterpress, foam-assisted stamping, and stamping. A stamped pattern’s printing parameters might vary based on the type of stamping technique utilized. It should be pointed out that the ink is cheap, and the stamp may be recyclable. Stamping is usually cost-effective and does not require expensive technologies such as laser scribing or photolithography. Since this printing technology can manufacture flexible devices with easy procedures, it provides a new resource for MSC device production [123].

**Figure 12:**
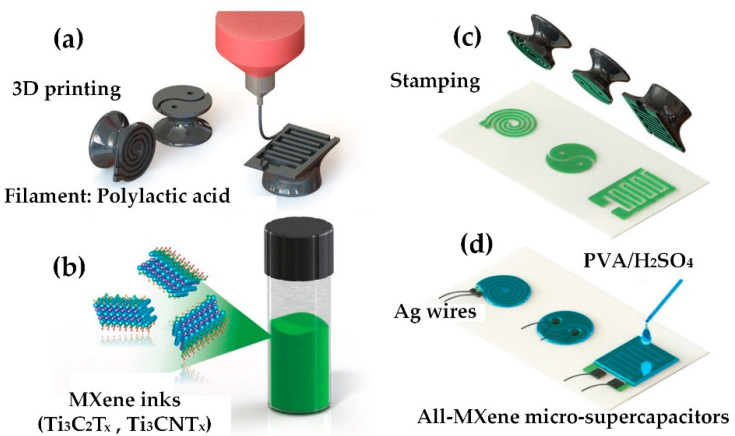
Fabrication of all-MXene-based MSCs using the stamping strategy. (**a**) stamps are first 3D printed, (**b**) MXene ink is controlled and prepared, (**c**) MXene ink is firmly pressed and stamped onto a rough hydrophilic substrate, and (**d**) upon attaching Ag wires and casting the electrolyte, the device is left to dry in air to form solid MXene-based MSC, reprinted with permission from [104].

Zhang et al. [104] developed a new stamping strategy, as shown in Figure 12, to 3D print a viscous water-based MXene ink to quickly prepare high-performance coplanar MXene MSC; using continuous thin-film nanostructures, these MSCs no longer have metal current collectors and may achieve a rapid electron transfer, thereby obtaining a high-speed response in solid-state devices. In addition, functionalized MXene imprinted on the hydrophilic porous paper also improves the accessibility of the MXene sheet to electrolyte ions. The combination of the pseudocapacitive behavior, high density and significant thickness of the Ti_3_C_2_T_x_ film may lead to a significant increase in the surface capacitance of each electrode. For example, the interdigitated MSC Ti_3_C_2_T_x_ has areal capacitances of 61 mF cm^−2^ and 50 mF cm^−2^, respectively, under 25 μA cm^−2^ and 800 μA cm^−2^. It was also presented that the production of MXene-MSCs can be increased by designing pads and cylindrical stamps, followed by a cold rolling method. Based on this method and the use of cold rolling and pressing, it is possible to produce dozens of MSCs with a high capacity of 56.8 mFcm^−2^ at 10 mV S^−1^ in a very short time. It is thought that by further optimizing the synthesis of MXenes, adjusting their particle size and chemical composition, and controlling surface functional groups, an MSC with greater surface/volume capacity and faster solid processing speed could be developed, opening up many exciting possibilities for the production of high-performance energy storage devices [104]. In all, rare studies on MXene stamping are available in the literature, and hence there still exist a lot of questions unanswered regarding the process parameters, materials, characterization and potential applications.

### 4.5. Patterned Coating

In spite of the advantages of printing in terms of resolution, scalability, and pattern complexity, equipment and ink properties often pose a challenge, limiting the number of materials that can be printed, not to mention even more complicated techniques to form printable inks from raw materials [75]. In this regard, traditional coating methods, e.g., drop-casting [125,126,127], spin coating [128,129,130], spray coating [131,132,133,134], vacuum filtration [125,135], etc. are ideal for testing materials and prototyping devices, as they have less stringent equipment and ink requirements. Because of the surface functional groups on MXene nanosheets, they can be processed in solution, which can be sprayed, spun, or dip-coated in order to make highly conductive thin films [136]. When compared to printing methods, patterned coatings are more cost-effective; however, the pattern complexity, tunable thicknesses, and surface roughness are much more challenging to manipulate with accuracy.

**Figure 13:**
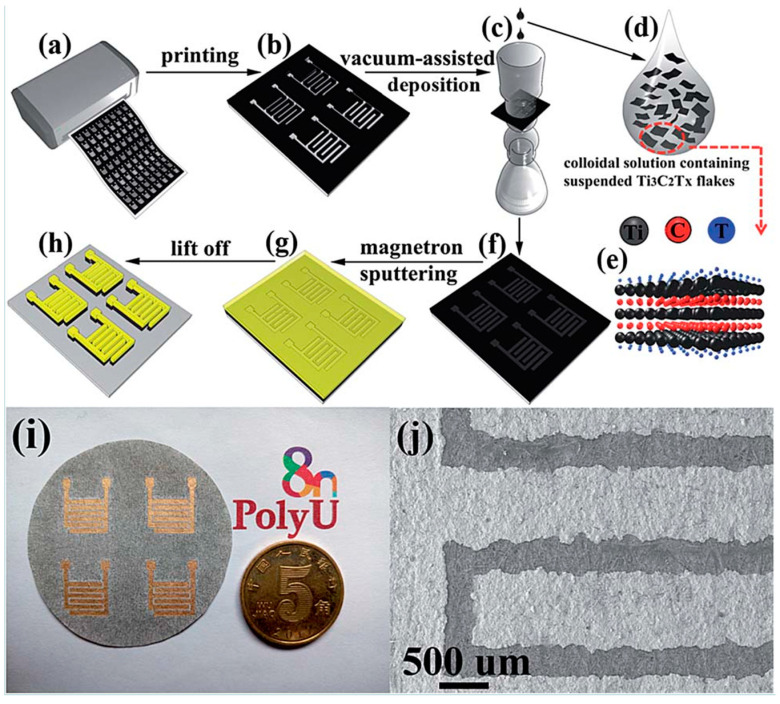
Flowchart of the easily manipulated protocol for patterning of fully delaminated few-layered MXene (Ti_3_C_2_T_x_) flakes on paper for planar symmetric MSCs: (**a**) a common laser printer for printing an interdigital circuit template designed with drawing software; (**b**) paper substrate with the laser-printed template; (**c**) vacuum-assisted deposition of fully delaminated few-layered MXene flakes on the printed paper to fabricate the electroactive layer at the bottom; (**d**) schematic of colloidal solution containing fully delaminated suspended fewlayered MXene flakes; (**e**) schematic of the crystal structure of Ti_3_C_2_T_x_ flakes (MXene); “T” symbolizes the surface atoms and atomic group, such as OH, F, and O; (**f**) the bottom binder/conductive-additive free electroactive layer based on few-layered Ti_3_C_2_T_x_ flakes; (**g**) magnetron sputtering of a top Au film that served as a current collector on the electroactive layer at the bottom; (**h**) final lift-off to form the MXene-based planar MSCs; (**i**) a photo of the finished MXene-based planar MSCs; (**j**) the corresponding SEM photo of the fabricated interdigital electrodes of the devices, reprinted with permission from [137].

Hu et al. [137] described an approach to creating coplanar and adjustable interdigital electrodes by patterning completely delaminated few-layered MXene flakes onto printed paper, as presented in Figure 13. A solid-state MSC’s volumetric capacitance may increase by at least 460 percent with increasing thickness of the MXene active layer, compared to advanced carbon-based planar symmetric MSCs (0.1–6 mF cm^2^). Devices with volumetric energy densities of 5.48 mWh cm^3^ or more are equivalent to or better than MSCs produced from other types of material, such as carbon or metallic oxides or paper-based polymers. High conductivity, fast intercalation, and hydrophilic surfaces are only a few of the great characteristics of MXene-based planar solid-state symmetric MSCs that exhibit good electrochemical performance. A major distinction is that this manufacturing procedure does not use any binder, conducting additives, or organic cosolvents. A unique, layered porous electrode structure has been created by Hu et al. [137] by aligning MXene flakes along the c-axis with no, or very minimal, binder/conductive additives. The electrode structure was highly electrically conductive due to the short ion diffusion distance between the electrode materials and electrolyte. Furthermore, by designing the electrode stacks so that the ions discharge rapidly, the resistance could be further decreased, allowing for the rapid diffusion of electrolytes. In light of these results, MXene-based symmetric MSCs may be suitable for use as miniaturized electronic devices [137].

**Figure 14:**
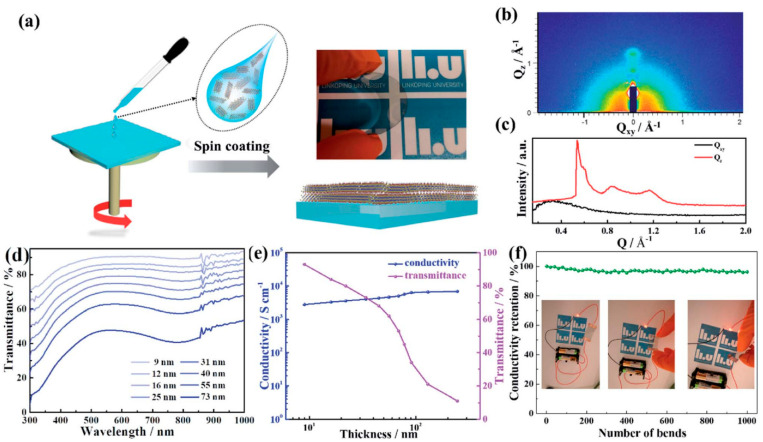
Optoelectronic properties of Ti_3_C_2_T_x_ films. (**a**) Schematic of the preparation of a transparent flexible electrode. (**b**) Two-dimensional GIWAXS pattern of Ti_3_C_2_T_x_ film prepared by the spin-coating method. (**c**) Out-of-plane and in-plane line-cut profiles from the GIWAXS pattern. (**d**) Transmittance spectra of Ti_3_C_2_T_x_ films of various thicknesses. (**e**) Variations in conductivity and transmittance of Ti_3_C_2_T_x_ electrodes as a function of thickness. (**f**) Conductivity retention with the number of bends for flexible Ti_3_C_2_T_x_ electrodes on PET substrates. The inset image shows the conductivity of the Ti_3_C_2_T_x_ electrodes in bent and twisted states, reprinted with permission from [136].

MXene films with high transmittance, conductivity and excellent mechanical stability are good candidates for transparent flexible electrodes for high-performance organic photovoltaic (OPV) [136]. A major draw of organic photovoltaics (OPVs) is their high flexibility, lightweight, low cost, and ease of printing for long-term renewable energy production. As a result of the electrical characteristics of MXenes, Qin et al. [136] have developed vertically stacked Ti_3_C_2_T_x_ MXene-based solid-state photovoltaic supercapacitors. A series of highly conducting, transparent and flexible films were produced, consisting of nanoflakes aligned parallel to the substrates, by spin-casting colloidal solutions of Ti_3_C_2_T_x_ nanosheets. On any substrate, spin-coating may produce smoother, more flexible transparent electrodes for OPVs by using tiny MXene flakes (Figure 14a). Ti_3_C_2_T_x_ spin-coated films displayed apparent arcs of diffracted intensity in the two-dimensional GIWAXS patterns (Figure 14b), indicating the development of regular orientations, being a substantial difference between the in-plane and out-of-plane diffraction signals, which indicates that flakes are aligned parallel to the substrate plane in the presence of shear force. In order to gain insight into the characteristics of Ti_3_C_2_T_x_ optoelectronic film, transmittance spectra with film thickness were measured (Figure 14d), wherein the transmittance reduced with increasing film thickness as well as a large visible peak. Spin-coated Ti_3_C_2_T_x_ films were measured for their transmittance at 550 nm and conductivity, and the results are shown in Figure 14e [136].

Sarycheva et al. [133] used 2D titanium carbide MXenein wireless communication devices due to their unique properties. They produced a clear MXene antenna through one step of spray coating. According to the results, a thickness of 100 nm and a reflection coefficient of less than 10 dB was obtained. It is also possible to achieve a reflection coefficient of -65 dB by increasing the thickness of the antenna to millimeter dimensions. In the study, titanium carbide MXene is found to operate below the skin depth of copper and other metals, presenting an opportunity to produce transparent antennas. Due to their high conductivity and water dispersibility, MXenes have been found to be an ideal material for the industrial production of various portable, flexible, and wearable electronic devices [133].

As opposed to other printing processes, they are less in demand and also less expensive. The disadvantage of this method is that there is no control over the thickness and roughness of the surface of the design, which is a major drawback. In addition, complex patterns will not be easy to achieve, unlike other methods [75].

## 5. Summary, Pitfalls, and Perspectives

MXenes have recently emerged as a revolutionary class of material displaying exceptional tailored-made properties. The onward journey and remarkable rise are establishing MXene-based materials as multifaceted playgrounds for technology-oriented explorations and are offering a tool-box for the ad hoc tailoring of advanced materials capable of effectively addressing current and future societal challenges. Unexpected applications have witnessed tremendous growth owing to the material’s unique chemical and physical properties, including, among others, optical, electrical, mechanical and thermal characteristics. Attaining an in-depth and critical understanding of the broadest arsenal of such unique and new properties, as well as the synergistic effects of the assorted characteristics, will play a pivotal role in new discoveries in both research and industrial sectors. The present paper aims to keep the readers updated with the recent developments in MXene-based inks, including their formulations, preparations and applications in a variety of fields ranging from energy and storage areas to biomaterials. Unlike other 2D sheets like graphene or dichalcogenides being well-implemented in real industrial applications such as batteries and electrodes, among others, the implementation of MXene inks in real industrial applications is still in its infancy stage and hence still, much attention should be made to advance the field and mature at different aspects ranging from fundamental sciences and manufacturing expenses to those practical challenges. It is also emphasized that unlike many other types of 2D materials, MXene has special traits such as mechano-ceramic behavior, hydrophilicity as well as rich surface chemistry, making them a promising additive in many liquid-based hybrid materials and structures. First of all, the basics regarding the MAX phases and the process of turning MAX to MXene nanoflakes have been proposed. The composition of MXene inks with the preparation of MXene-based inks has then been introduced. Due to their vital importance, the stability of MXene nanosheets in different media has been discussed, and then considerable attention is paid to the fluidity and rheological properties and features of the MXenes, being essential parameters dictating the final quality and performance of the MXene ink. Further, different printing techniques ranging from inkjet printing and stamping to 3D printing, are elaborated to discuss the current state of the art in this ever-growing field. Finally, despite the brilliant progress made, there are still multiple longstanding hurdles that need to be overcome, so it is believed that still, innumerable questions are to be answered to fill the existing voids. For instance, MXene synthesis with controllable surface terminations is one of the major challenges ahead. The uniformity of these functional groups effectively improves the rheological properties and colloidal stability of MXene inks and also changes the final print quality. Regarding computational study, rare studies are available in this field, and much more attention is needed to explore ink behavior in different situations. Further, few types of MXene (almost always Ti_3_C_2_T_x_) have been used in inks and subsequent printing techniques, and it is strongly thought other different MXenes also have different features when hybridized in varied inks. Most of the current research has been undertaken in the field of energy storage and electrodes for capacitors; however, other 2D nanomaterials have been used in a variety of research and industrial sectors with promising results. It is believed a great variety of transition metals with various surface terminations makes it possible to achieve new compounds for specific and unique applications. Printing of heterogeneous structures, including MXene and other two-dimensional nanomaterials, could also be considered in the future. Finally, as one of the most challenging issues, MXene inks have relatively low stability and oxidation resistance in different materials. In order to commercialize MXene-based inks, there is a long way to go for scalability, longer shelf life, higher performance and functionality. 

## Data Availability

All data is contained within the article.
